# Aldehyde–Aminotriazole Condensation Products as Novel Corrosion Inhibitors for Mild Steel in Hydrochloric Acid

**DOI:** 10.3390/polym17202761

**Published:** 2025-10-15

**Authors:** Daniil R. Bazanov, Yaroslav G. Avdeev, Tatyana A. Nenasheva, Andrey Yu. Luchkin, Dmitrii M. Mazur, Yury B. Makarychev, Tatiana E. Andreeva, Andrey I. Marshakov, Yurii I. Kuznetsov

**Affiliations:** 1 Department of Chemistry, Shenzhen MSU-BIT University, Shenzhen 518172, China; 2 Department of Chemistry, M. V. Lomonosov Moscow State University, 119991 Moscow, Russia; 3 Frumkin Institute of Physical Chemistry and Electrochemistry, Russian Academy of Sciences, 31-4, Leninsky Prospect, 119071 Moscow, Russia

**Keywords:** corrosion, steel, hydrochloric acid, corrosion inhibitors, unsaturated aldehydes, cinnamaldehyde, aminotriazole

## Abstract

The significance of this study arises from the urgent need to develop new corrosion inhibitors for the oil and gas industry. These inhibitors should be synthesized from readily available raw materials and be capable of providing effective protection for steel structures against corrosion when exposed to technological hydrochloric acid solutions over a wide temperature range (20–100 °C). The search for such environmentally acceptable and cost-efficient inhibitors is crucial for improving the durability and operational safety of oilfield equipment under aggressive acidic conditions. A new high-temperature corrosion inhibitor for steel in hydrochloric acid solutions has therefore been developed. The inhibitor, designated CATA, is the product of chemical condensation between cinnamaldehyde and 3-amino-1,2,4-triazole. Its protective action is based on the formation of an organic layer up to 12 nm thick, strongly bound to the steel surface. The results suggest with high probability that this protective film consists of polymeric products formed through chemical transformation of CATA on the corroding metal surface. It was shown that the addition of CATA significantly suppresses the electrode processes of steel, affecting both cathodic and anodic partial reactions as well as the kinetics of hydrogen permeation. Adsorption of CATA on steel is satisfactorily described by the Temkin isotherm. The free energy of adsorption (−Δ*G_ads_*) was determined to be 54 kJ mol^−1^, which is characteristic of chemisorption. This unique inhibition mechanism enables effective corrosion protection of steel in HCl solutions over a wide temperature range (20–100 °C). Under the most aggressive experimental conditions (2 M HCl, 100 °C), the addition of 10 mM CATA achieved an inhibition efficiency of 99.6%, with a corrosion rate of 3.3 g m^−2^ h^−1^, which represents an outstanding result. Furthermore, for spring steels, even in hot HCl solutions (20–60 °C), CATA strongly suppresses hydrogen uptake and allows complete preservation of their ductility.

## 1. Introduction

The potential for increasing production of liquid and gaseous hydrocarbons in the oil and gas industry is strongly associated with carbonate reservoirs, which are estimated to contain up to 60% of the world’s oil and natural gas reserves [1]. Currently, more than half of global hydrocarbon production comes from such formations. For over a century, hydrochloric acid (HCl) stimulation of the near-wellbore zone in carbonate reservoirs has remained one of the primary methods for enhancing hydrocarbon recovery [2,3,4,5]. However, the direct use of aqueous HCl solutions—highly aggressive toward steels—poses serious risks, as it accelerates corrosion of downhole metallic equipment and surface treatment facilities. The most effective preventive measure against acid-induced corrosion is the application of corrosion inhibitors (CIs) [6,7].

A wide variety of CIs have been developed for protecting steels in hydrochloric acid solutions [6,7,8,9,10,11]. Industrially relevant inhibitors must remain effective over a broad temperature range (25–100 °C), since carbonate reservoirs often contain hydrocarbons at elevated temperatures (up to 100 °C) [4], where the aggressiveness of HCl is markedly intensified.

Recent publications have reported studies aimed at identifying new substances capable of slowing down the corrosion of steels in hydrochloric acid solutions (Table 1). As corrosion inhibitors for steels in such environments, mainly synthetic compounds [12,13,14,15,16], plant extracts [17,18,19,20], and pharmaceutical drugs [21] have been investigated. The data presented for these inhibitors generally refer to their protective performance at temperatures not exceeding 60 °C. Moreover, for plant extracts, the protective efficiency of the metal decreases significantly with increasing temperature of the aggressive medium. Based on the analysis of the available literature, it can be concluded that there are certain difficulties in developing a corrosion inhibitor for steel in hydrochloric acid that can provide effective protection over a wide temperature range (20–100 °C), which is required for the practical use of hydrochloric acid solutions in oil and gas production. The development of such a steel corrosion inhibitor represents an innovative aspect of this study, as its protective performance should surpass that of the newly reported inhibitors described in the literature.

Among the most promising organic inhibitors for hot HCl media are unsaturated carbonyl compounds [22], particularly aldehydes [23,24,25,26,27,28,29,30,31,32] and ketones [33,34]. Cinnamaldehyde (CA) is one of the best-studied representatives of this group. In 2 M HCl at 60–80 °C, CA at 10 mM achieves a corrosion protection efficiency (Z) of 89.1–93.9% for mild steel [31]. Its performance can be further enhanced by co-additives such as N-dodecylpyridinium bromide [23]. The inhibitory action of CA is attributed to its conjugated C=C and C=O bonds, which facilitate polymeric film formation on the steel surface during acid-induced molecular transformations [30,31,32]. CA is attractive not only as an inhibitor but also as a renewable feedstock, being obtainable synthetically from acetaldehyde and benzaldehyde [35] or by processing natural sources such as *Cinnamomum verum* (True cinnamon) and *Cinnamomum cassia* (Cassia cinnamon) [36,37].

Aldehydes readily undergo condensation with amines to form azomethines, many of which display strong anticorrosion properties in acidic media. For instance, the condensation product of chitosan and CA (600 mg/L) provides 87.9% protection for mild steel in 15% HCl at 25 °C, which increases to 92.7% upon addition of 10 mM KI [38]. Similarly, 200 ppm of N′-(3-phenylallylidene)isonicotinohydrazide in 1 M HCl (25 °C) yields Z values up to 99.5% [39]. Other reported azomethines achieve efficiencies of 96–98% under comparable conditions, as shown by EIS and voltammetric studies [40,41].

Building on these findings, we propose the synthesis of a new CI by condensing CA with 3-amino-1,2,4-triazole (TA). Triazole derivatives are known to suppress steel corrosion in HCl solutions across wide temperature ranges, primarily due to their ability to chemisorb and form stable polymolecular protective layers [42]. The condensation of CA with TA is expected to yield a mixture of 1,2- and 1,4-adducts [43], containing both C=C and C=N bonds that could undergo polymerization on the steel surface to form a robust protective film. The triazole moiety should further promote chemisorption. Importantly, we focus on the mixed condensation product as a whole, rather than on isolating individual components, since separation would significantly increase production costs.

## 2. Materials and Methods

### 2.1. Materials

The effect of the studied organic compounds on the corrosion behavior of mild steel St3 (wt.%: 0.14–0.22 C; 0.15–0.30 Si; 0.40–0.56 Mn; up to 0.30 Ni; up to 0.30 Cr; up to 0.30 Cu; up to 0.08 As; up to 0.05 S; up to 0.05 P; up to 0.008 N) (RU) in HCl solutions was investigated using rectangular samples (50 × 20 × 3 mm). The effect of the tested corrosion inhibitors on hydrogen absorption during corrosion and on the preservation of mechanical properties was studied using hydrogen-embrittlement-sensitive spring steel 70S2KhA (wt.%: 0.65–0.75 C; 1.4–1.7 Si; 0.40–0.60 Mn; 0.20–0.40 Cr; up to 0.25 Ni; up to 0.20 Cu; up to 0.025 S; up to 0.025 P) in rectangular specimens (110 × 8.0 × 0.5 mm). The exposure time of the metal samples to the aggressive medium was 2 h.

Voltammetric and EIS measurements were carried out on the end surfaces of cylindrical St3 steel samples embedded in Teflon, with working surface areas (S) of 0.72 and 0.50 cm^2^, respectively. To study hydrogen permeation kinetics, palladium-coated membranes of mild steel 08kp (wt.%: 0.05 C; 0.38 Mn; 0.16 Al; 0.15 Cu; 0.09 Ni; 0.05 Cr; 0.04 S; 0.035 P; 0.03 Si) were used, with a thickness of 0.100 mm and a working surface area of 4.25 cm^2^.

Working hydrochloric acid solutions for corrosion and electrochemical studies were prepared from distilled water and concentrated 36% HCl (“chemically pure” grade). Corrosion tests were carried out in naturally aerated HCl solutions, whereas electrochemical experiments were performed in solutions deaerated with argon gas (“chemically pure” grade).

As corrosion inhibitors, both individual organic compounds (Table 2) and their condensation products were investigated. All aldehydes and 3-amino-1,2,4-triazole were purchased from Macklin, China. By mixing correspondent aldehydes with 3-amino-1,2,4-triazole (TA) in a 1:1 molar ratio, three condensation products were obtained: cinnamaldehyde with aminotriazole (CATA), benzaldehyde with aminotriazole (BATA), and crotonaldehyde with aminotriazole (CrATA). Condensation reactions were carried out in ethanol (50%) at room temperature. After completion, the solvent was removed by evaporation, and, in cases where a precipitate formed, it was separated by filtration.

The condensation products of aldehydes with TA are mixtures of various organic compounds, mainly 1,2- and 1,4-condensation products, which complicates precise quantification and preparation of solutions with accurately defined concentrations. Assuming that aldehyde molecules do not undergo significant self-reactions during condensation, and that TA reacts completely with aldehyde molecules, the number of product molecules formed is equal to the number of initial aldehyde molecules. Although approximate, this assumption allows a valid comparison of the protective efficiencies of different condensation products and their precursor compounds. For comparison between condensation products and mixtures of individual components, solutions were prepared in which the inhibitor concentration equaled the concentrations of the corresponding aldehyde and TA. In this study, the baseline concentration of both individual organic compounds and condensation products in the corrosive medium was 10 mM.

All inhibitors were introduced into the test corrosive media in the form of ethanol solutions, with the final ethanol content in the media amounting to 1.2 mol L^−1^.

### 2.2. Methods

#### 2.2.1. Determination of Corrosion Rate and Inhibition Efficiency

The corrosion rate of steel specimens was determined gravimetrically from the change in their mass (Δ*m*, g) before and after 2 h exposure to the corrosive medium. The specific mass loss was calculated as:

Δ*m*^s^ = Δ*m S*^−1^, (1)

where *S* is the surface area of the specimen (m^2^). The corrosion rate (g m^−2^ h^−1^) was then determined as:

*W* = Δ*m*^s^ *τ*^−1^, (2)

where τ is the exposure time.

The inhibition efficiency (Z, %) of corrosion inhibitors was calculated from the mass loss data as:

Z = [(Δ*m*^s^_0_ − Δ*m*^s^_in_) (Δ*m*^s^_0_)^−1^]∙100%, (3)

where Δ*m*^s^_0_ and Δ*m*^s^_in_ are the specific mass losses of steel in the corrosive medium without and with the addition of the inhibitor, respectively.

#### 2.2.2. Vacuum Extraction Method

The amount of hydrogen absorbed by steel during exposure to the aggressive medium was determined using the vacuum extraction method. In this approach, the specimen was heated to 500 °C and the hydrogen released into the evacuated part of the setup was measured. The pressure increase due to hydrogen desorption was monitored with a McLeod mercury manometer. The specific hydrogen content in steel (mL (100 g of metal)^−1^) was calculated as:

*V*^s^(H_2_) = *G* Δ*p*(H_2_) *m*^−1^, (4)

where *G* is an empirical constant determined by the apparatus, Δ*p*(H_2_) is the pressure increase caused by hydrogen desorption, and m is the mass of the specimen.

To evaluate the ability of corrosion inhibitors to suppress hydrogen absorption, tests were performed on hydrogen-embrittlement-sensitive spring steel 70S2KhA. The reported hydrogen contents are corrected for the background hydrogen level in the investigated steel batch, which was 0.34 mL (100 g of steel)^−1^.

#### 2.2.3. Cyclic Bending Tests

Hydrogen absorption by spring steels results in a loss of mechanical properties, which indirectly reflects the presence of dissolved hydrogen. The retention of mechanical performance of spring steel 70S2KhA after corrosion in acidic solutions was therefore assessed by the cyclic bending endurance of strip specimens. The retention coefficient (π) was calculated as:

*π* = *β*_0_
*β*^−1^ 100%, (5)

where β_0_ is the number of bends sustained by the as-received strip specimens, and β is the number of bends after exposure to the acidic solution.

#### 2.2.4. Voltammetry

The effect of the studied inhibitors on the electrode reactions of St3 steel was examined by voltammetry. Measurements were carried out in a thermostated glass electrochemical cell equipped with a separate compartment for the reference and auxiliary electrodes. The working electrode was St3 steel, the reference electrode was a saturated KCl Ag/AgCl electrode, and the auxiliary electrode was a platinum plate (S = 2.0 cm^2^).

The working electrode was prepared by grinding with abrasive paper, polishing with diamond paste (ASM 0.5/0), and rinsing successively with ethanol and acetone. Polarization curves were recorded using an IPC-PRO MF potentiostat (Cronas Ltd., Moscow, Russia) at a scan rate of 0.5 mV s^−1^, in the potential range −0.4–0 V. All potentials are reported versus the standard hydrogen electrode (SHE).

#### 2.2.5. Electrochemical Impedance Spectroscopy (EIS)

EIS studies were performed using the IPC-PRO MF potentiostat equipped with a frequency response analyzer, employing the same electrochemical cell described in Section 2.2.4. Measurements were carried out on cathodically polarized steel electrodes (E = −0.30 V). The impedance spectra were recorded in the frequency range 0.010–3000 Hz with an AC perturbation amplitude of 20 mV.

The surface coverage by the organic compound (*θ*_in_) was determined according to the relation:

(6) $$\theta_{in}=\frac{C_{\mathrm{dl}}^{0}-C_{\mathrm{dl}}}{C_{\mathrm{dl}}^{0}-C_{\mathrm{dl}}^{\infty }}\;,$$

where $C_{\mathrm{dl}}^{0}$ and $C_{\mathrm{dl}}$ are the double-layer capacitances of the working electrode in the absence and presence of the organic additive, respectively, and $C_{\mathrm{dl}}^{\infty }$ is the capacitance under conditions of complete adsorption of the organic substance on steel.

The free energy of adsorption of the organic compound (−Δ*G_ads_*) on steel from HCl solution was calculated using the Temkin monolayer adsorption isotherm, which is commonly employed to describe the adsorption of nitrogen-containing inhibitors on steel in acidic solutions [44]:

*θ*_in_ = *f*^−1^ ln[*B*_ads_
*C*_in_], (7)

where *θ_in_* is the degree of surface coverage by the organic compound, *f* is the heterogeneity factor of the metal surface, *B_ads_* is the adsorption equilibrium constant, and *C_in_* is the inhibitor concentration in the corrosive medium. Equation (7) assumes a linear decrease in adsorption energy with increasing surface coverage. This behavior arises from either the intrinsic heterogeneity of the sorbent surface or induced heterogeneity resulting from interactions among adsorbed particles.

The adsorption free energy (−Δ*G_ads_*) was then calculated from *B_ads_* as:

−Δ*G*_ads_ = *R T* ln [55.5 *B*_ads_] (8)

#### 2.2.6. Bipolar Electrode Method

**Figure 1:**
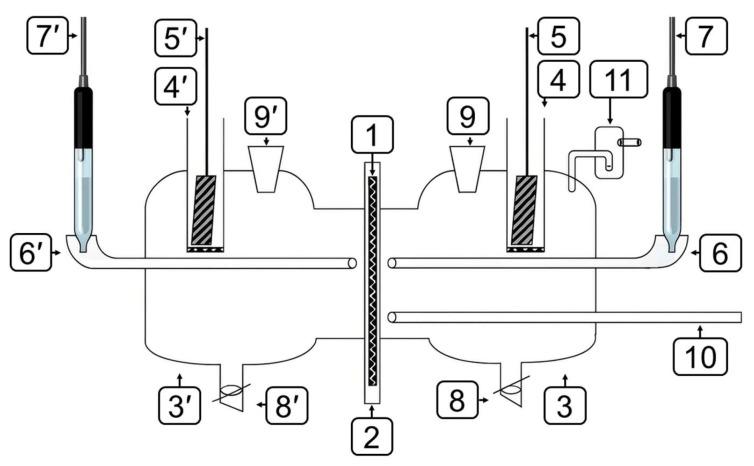
Devanatkhan-Stakhursky electrochemical cell. 1—working electrode (membrane); 2—Teflon holder; 3—working part of the cell; 3′—diffusion part of the cell; 4, 4′—auxiliary electrode cell; 5, 5′—auxiliary electrode; 6, 6′—electrolytic switch; 7, 7′—reference electrode; 8, 8′—tap for draining the solution; 9, 9′—solution input into the cell; 10—argon input into the cell; 11—water seal.

Electrochemical measurements were performed in a two-compartment Devanathan–Stachurski cell (Figure 1) [45]. The dependencies of the cathodic hydrogen evolution rate, anodic metal dissolution rate, and hydrogen permeation rate on potential were determined. The preparation of the working electrode and the detailed experimental procedure are described in [46].

#### 2.2.7. IPZ Analysis Method

To calculate the rate constants of the main stages of cathodic hydrogen evolution and hydrogen penetration into steel, as well as the amounts of hydrogen absorbed and adsorbed by the metal in both blank solutions and inhibitor-containing media, the IPZ analysis method was employed [46,47,48].

Taking into account the adsorption of atomic hydrogen and inhibitor particles on the metal surface, the discharge reaction of hydrogen ions in acidic media can be described as follows:

*i*_c_ = *Fk*_c_ [(1 − *θ*_in_)*^r^*^1^ − *θ*_H_] exp(−α*FE*/*RT*), (9)

where *F* is the Faraday constant, *k*_c_ the rate constant of hydrogen ion discharge,

*θ_in_* the surface coverage by the inhibitor, *r*_1_ the number of adsorption sites occupied by a hydrogen ion on the surface, *θ*_*H*_ the surface coverage by hydrogen, *α* the transfer coefficient of the hydrogen ion discharge reaction, *R* the gas constant, and *T* the absolute temperature.

The rate of the chemical recombination of H atoms (*iᵣ*) is determined as

(10) $$i_{r}=Fk_{r}\theta_{H}^{2},$$

where *kᵣ* is the rate constant of hydrogen atom recombination.

The rate of hydrogen penetration into the metal (*iₚ*) and its steady-state diffusion in the membrane are described by the following relations:

(11) $$i_{p}=F\left(k_{abs}\theta_{H}-k_{des}C_{H}^{s}\right)$$

(12) $$i_{p}=\frac{FDC_{H}^{s}}{L}$$

where *k_abs_* and *k_des_* are the rate constants of hydrogen absorption and desorption from the metal phase, $C_{H}^{s}$ the concentration of diffusible hydrogen in the metal phase, *L* the membrane thickness, and *D* the diffusion coefficient of hydrogen in the metal.

From Equations (11) and (12), it follows that:

(13) $$\theta_{H}=\frac{k_{des}+\frac{D}{L}}{k_{abs}}C_{H}^{s}=kC_{H}^{s}$$

where *k* is the kinetic–diffusion constant that characterizes the relationship between the hydrogen atom concentration at the surface and in the metal phase.

Using Equations (9)–(12) under steady-state conditions (*i*_c_ = *i*_p_ + *i*_r_), one can obtain [47,48]:

(14) $$i_{c}\exp\left(\frac{\alpha FE}{RT}\right)=Fk_{1}a_{H^{+}}{\left(1-\theta_{inh}\right)}^{r1}-\frac{k_{1}a_{H^{+}}kL}{D}\cdot i_{p}$$

(15) $$i_{p}=\frac{D\sqrt{F}}{Lk\sqrt{k_{r}}}\cdot \sqrt{i_{c}-i_{p}.}=\frac{D\sqrt{F}}{Lk\sqrt{k_{r}}}\sqrt{i_{r}}$$

By combining Equations (9), (10), (12), and (13), an expression is derived for calculating the degree of steel surface coverage by hydrogen:

(16) $$\theta_{H}=\frac{-\left(k_{1,i}+\frac{D}{Lk}\right)+\sqrt{{\left(k_{1,i}+\frac{D}{Lk}\right)}^{2}+4k_{r}k_{1,i}{\left(1-\theta_{inh}\right)}^{r1}}}{2k_{r}}\cdot$$

(17) $$k_{1,i}=k_{1}a_{H^{+}}\exp\left(\frac{\alpha FE_{i}}{RT}\right)$$

where $k_{1,i}$ is the formal rate constant of hydrogen ion discharge at potential *Eᵢ*, and $a_{H^{+}}$ the hydrogen ion activity.

A more detailed description of the IPZ analysis method is given in [46].

#### 2.2.8. AFM Method

Surface topography maps of the steel samples were obtained using a SolverNexT II atomic force microscope (NovaPhotonix LLC, St. Petersburg, Russian Federation) in Kelvin Probe Force Microscopy (KPFM) mode with phase modulation. A cantilever with a conductive WC_2_ coating, having a resonance frequency of 57.42 kHz, was employed. Image processing was performed using Gwyddion 2.61 [49]. The work function was calculated by subtracting the average surface potential of the sample from the probe surface potential (5.572 eV), which was determined using freshly cleaved pyrolytic graphite.

The samples were polished using special diamond pastes with grain sizes up to 0.5 μm. Final polishing was carried out with a colloidal silica suspension with a particle size of 0.05 μm (O.P.S., Struers GmbH, Willich, Germany). After polishing, micrographs and surface topography maps were obtained.

#### 2.2.9. XPS Method

Quantitative and qualitative analysis of the surface layers formed by organic compounds on St3 steel specimens in HCl solutions was performed using X-ray photoelectron spectroscopy (XPS). The measurements were carried out on an HV100 Auger microscope (VG, London, UK) equipped with a chamber for XPS spectrum acquisition. The pressure in the analytical chamber was maintained at better than 10^−9^ Torr. An Al anode operated at 200 W was used as the excitation source. The analyzer pass energy was set to 50 eV. Square St3 steel specimens (10 mm × 10 mm), prepared in the same manner as for corrosion tests, were used in the study.

The binding energy (*E_b_*) of photoelectrons ejected from the inner shells of atoms was calibrated against the C1s peak (285.0 eV), which originates from adventitious carbon layers formed by adsorbed diffusion pump oil vapors. Instrument calibration was verified using reference metallic Au and Cu samples cleaned by Ar^+^ ion sputtering (*E_b_*(Au4f_7/2_) = 84.0 eV and *E_b_*(Cu2p_3/2_) = 932.7 eV). Characteristic peaks for the following elements were measured: C1s, O1s, Fe2p, N1s, and Cl2p. Photoionization cross sections reported in [50] were used for quantitative evaluation. Background subtraction was performed using the Shirley method [51], and observed peaks were fitted with Gaussian functions including a Lorentzian contribution. Integral peak intensities were obtained, and protective film thicknesses were determined from the integrated peak areas of C1s, O1s, Fe2p, N1s, and Cl2p.

It should be noted that the quantification of iron depends strongly on the processing of Fe2p XPS spectra, which are complicated by multiple oxidation states and satellite lines, making intensity evaluation difficult [52,53]. Even in simpler cases, subtraction of the inelastic background, which extends toward higher binding energies, may lead to errors [54]. To address this, one iteration of background subtraction was performed to obtain the theoretical intensity ratio of the Fe2p doublet (2p_3_/_2_ to 2p_1_/_2_). In contrast, for the C1s, N1s, Cl2p, and O1s spectra, a second iteration did not alter the integrated peak areas.

Information on the thickness of the surface layers was obtained using the MultiQuant software (v. 1.2) [55], employing the photoionization cross sections reported by Scofield [56]. Inelastic mean free paths (IMFPs) of electrons were calculated using the approach proposed by Cumpson and Seah [57].

Protective films on steel surfaces were formed by immersing the metal specimens in HCl solutions containing the organic compound under study for 2 h. To remove weakly adsorbed organic molecules, the steel samples were treated in an additive-free 2 M HCl solution in an ultrasonic bath (three cycles of 6 min each). Parallel XPS studies were performed on steel surfaces after adsorption of the organic compound in HCl solution with and without ultrasonic cleaning. To determine the depth distribution of chemical elements and the thickness of the protective layer, argon ion sputtering of the steel surface was employed.

#### 2.2.10. Mass Spectrometry

A small portion of the obtained reaction mixture (10 mg) was dissolved in 1.5 mL of acetonitrile, diluted tenfold, and transferred into a 2 mL vial for subsequent analysis. Electrospray ionization (ESI) mass spectra were recorded using a TSQ Endura mass spectrometer (Thermo Fisher Scientific, Waltham, MA, USA). The sample solution was directly introduced into the ionization source with a syringe pump at a flow rate of 10 μL/min. Spectra were acquired for 90 s in the *m*/*z* range of 200–600 in positive ionization mode with a spray voltage of 3.4 kV. The ion optics capillary temperature was set to 275 °C, and the vaporizer temperature was maintained at 40 °C. Source gas parameters were: sheath gas—6 arbitrary units; and auxiliary gas—5 arbitrary units.

For tandem mass spectrometry (MS/MS) experiments, a triple quadrupole system was employed. The first quadrupole was used to isolate the parent ion of interest (isolation width 0.4 Da). Fragmentation was induced with argon at a collision cell pressure of 1 Torr. The collision energy was varied in the range of 10–30 arbitrary units. The third quadrupole was operated in scanning mode to detect all possible fragment ions. The system was controlled using Xcalibur software (v. 3.0), which was also applied for data acquisition and processing.

#### 2.2.11. Molecular Dynamics Simulations

Molecular dynamics (MD) simulations were carried out using BIOVIA Materials Studio 2023. The simulated cell consisted of a six-layer Fe(–100) surface, an organic molecule surrounded by 200 water molecules, and a vacuum layer introduced to avoid interactions between the water layer and the periodic continuation of the metal surface. The simulations were performed in the Forcite module of BIOVIA Materials Studio 2023 under the NVT ensemble at 65 °C, with the Andersen thermostat applied. A time step of 1 fs and a total simulation time of 100 ps were used. The COMPASSIII force field was employed for all calculations.

#### 2.2.12. Quantum-Chemical Calculations

The calculation of energy values, geometry optimization, and orbital visualization were performed using the Schrödinger 2023.1 software package (Jaguar module). The B3LYP functional with the QM Basic 6–31G** basis set was employed, with the solvent effects taken into account using the PBF implicit solvation model (water).

## 3. Results and Discussion

### 3.1. Effect of Aldehyde Condensation Products on Steel Corrosion in HCl Solution

Mild steel is highly susceptible to dissolution in 2 M HCl (Table 3). The specific weight loss and the corrosion rate of steel increase with temperature (t). An increase in t from 20 to 100 °C accelerates the corrosion process by a factor of 510. Among the tested condensation products, CATA demonstrated the most promising results: within the investigated temperature range, the corrosion rate did not exceed 3.3 g m^−2^ h^−1^, which is a remarkable outcome. Notably, the inhibitory efficiency of CATA improved with increasing temperature, allowing it to be classified as a high-temperature corrosion inhibitor [58]. As expected, the precursors (CA and TA) used for the synthesis of CATA were less effective in retarding corrosion than the final condensation product. Interestingly, in cold acidic solutions (20–40 °C), a mixture of 10 mM CA + 10 mM TA provided better protection than 10 mM CATA. However, in more aggressive, heated solutions, CATA exhibited superior inhibitory performance despite its lower effective concentration in the corrosive medium. This effect can be attributed to the higher total concentration of organic species in the presence of CA + TA, while at elevated temperatures, the stability and adsorption behavior of CATA ensured more effective protection.

The protective efficiency of the condensation products decreased in the following order: CATA > CrATA > BATA. The superior inhibitory effect of CATA can be related to its structural features, namely the presence of C=C and C=N bonds as well as a phenyl fragment, which enhance adsorption on the steel surface. In contrast, the inhibitory effects of CrATA and BATA were less pronounced compared to CATA, and in fact lower than those of their precursors CrA and BA, respectively. Thus, unlike the distinct synergistic effect observed for CATA relative to CA + TA, no such enhancement was evident for CrATA or BATA compared to their respective precursor mixtures.

**Figure 2:**
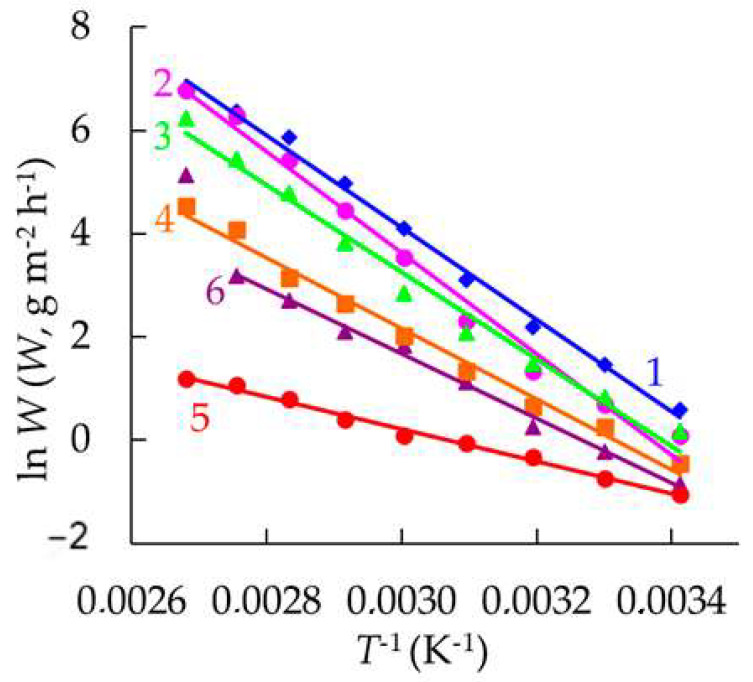
Dependence of ln *W* on T^−1^ for the corrosion of mild steel in 2.0 M HCl (1) and in the presence of TA (2), BATA (3), CrATA (4), CATA (5), and CA (6). C_in_ = 10 mM.

The temperature dependence of the corrosion rate of steel in HCl solutions containing the studied organic compounds was further analyzed using the Arrhenius equation:

*W* = *W*′ exp (−*E*_ac_ *R*^−1^*T*^−1^), (18)

where *W*′ is the pre-exponential factor, *E_ac_* is the effective activation energy of the corrosion process, R is the universal gas constant, and T is the absolute temperature. Among the tested inhibitors, CATA exerted the most pronounced effect on the activation energy of the corrosion process compared with the uninhibited medium (Table 4, Figure 2). The measured activation energy in its presence was 26 kJ mol^−1^. This relatively low value indicates a significant contribution of the diffusion-controlled stage to the corrosion process, consistent with the formation of a protective inhibitor layer on the steel surface that introduces diffusional limitations for the transport of protons to the metal surface and/or for the removal of corrosion products.

### 3.2. Effect of CATA and CA on the Mechanical Properties of Steel and Its Hydrogen Uptake

The addition of 10 mM CATA reduces the corrosion rate of spring steel in HCl solution within the temperature range t = 25–60 °C (Table 5). At the same time, this additive significantly suppresses both the absorption of hydrogen by the corroding metal and the associated loss of its mechanical properties. It should be emphasized that these results were obtained on spring steel, which is prone to absorbing the evolving hydrogen. In the case of mild steel, the hydrogen uptake in the presence of this condensation product is expected to be substantially lower. Under similar conditions, CA, which serves as the precursor in the synthesis of CATA, shows lower efficiency compared to the latter.

### 3.3. Effect of CATA on the Electrode Reactions of Steel

**Figure 3:**
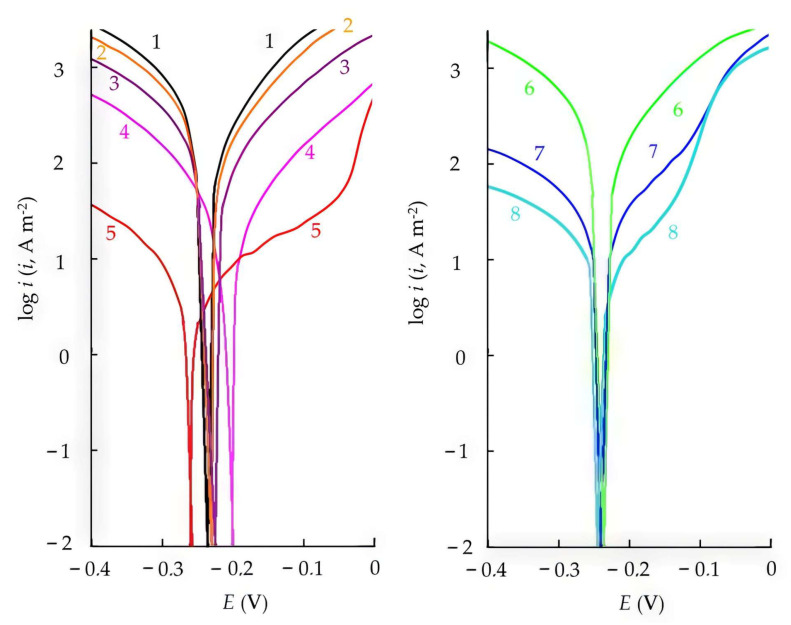
The polarization curves of mild steel (60 °C) in 2.0 M HCl (1) with additions of 0.03 mM CATA (2), 0.1 mM CATA (3), 1.0 mM CATA (4), 10.0 mM CATA (5), 10.0 mM TA (6), 10.0 mM CA (7), 10.0 mM CA + 10.0 mM TA (8).

The polarization behavior of steel in HCl solution and the parameters of the electrode reactions are presented in Table 6 and Figure 3. It can be seen that the influence of CATA on the electrode reactions of steel already appears at concentrations as low as C = 0.03 mM. This compound retards both electrode reactions of the metal. At C = 10 mM, the inhibitory effect of CATA on the electrode reactions significantly surpasses that of its precursors—CA, TA, and the CA + TA mixture. In the presence of CATA, high values of the Tafel slopes for both cathodic and anodic polarization curves are observed, indirectly indicating the formation of protective layers of the inhibitor on the steel surface, which ensure maximum efficiency in suppressing both the cathodic and anodic reactions. Thus, the high protective efficiency of CATA in retarding steel corrosion in HCl solutions results from its effective suppression of both electrode reactions of the metal (Zc = 99%, Za = 98.3%).

### 3.4. Adsorption of CATA on Steel

**Figure 4:**
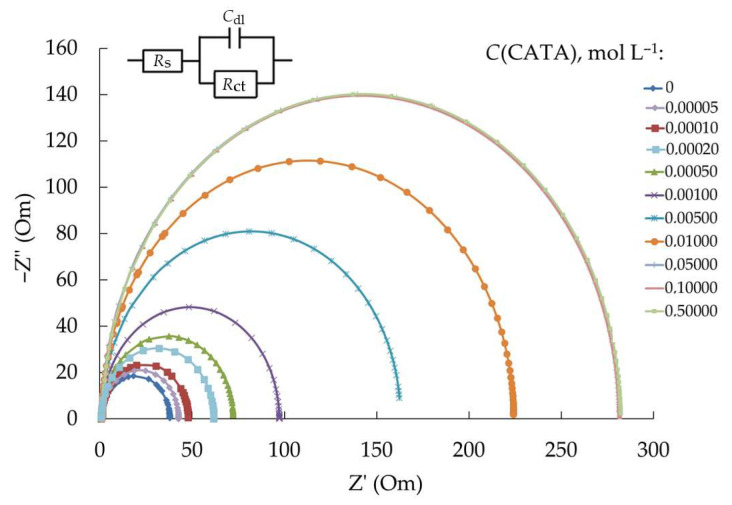
Nyquist plots of steel in 2.0 M HCl solution at 20 °C.

Among the investigated chemical compounds, CATA demonstrates the most significant corrosion-inhibiting effect on steel in HCl solution. Therefore, it is particularly important to study the adsorption of this compound on steel from the acid solution, as this is crucial for understanding the mechanism of its protective action. The Nyquist plots of the steel electrode in 2 M HCl solutions containing CATA additives are represented by semicircles close to ideal ones (Figure 4). An equivalent circuit that satisfactorily describes such an electrochemical system is a series–parallel electrical circuit composed of a capacitor (C_dl_, a frequency-independent capacitance often identified with the capacitance of the metal/electrolyte double layer) and resistances (R_ct_, the impedance associated in this system with the electrochemical reduction of protons on the metal surface, and R_s_, the solution resistance).

**Figure 5:**
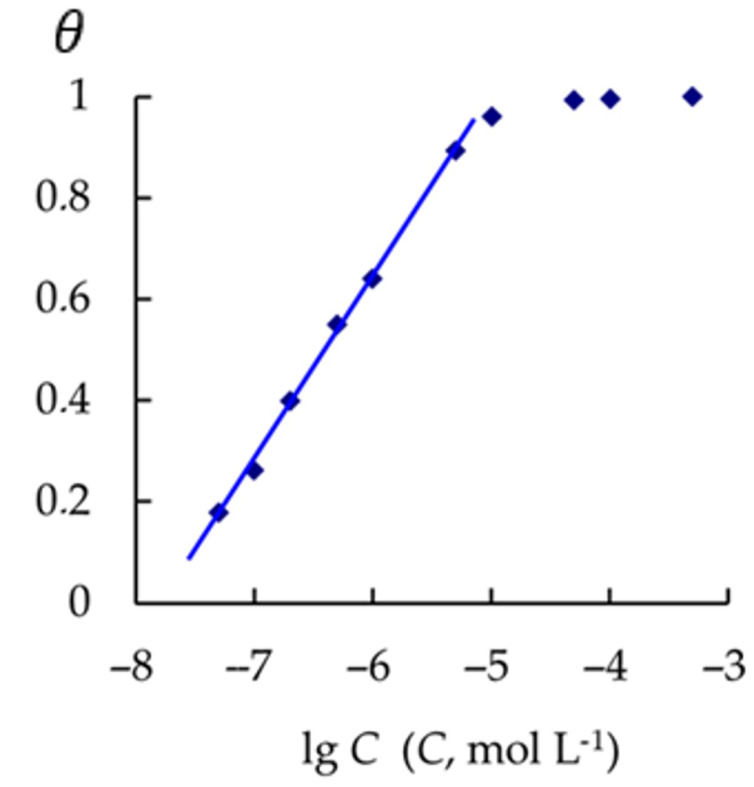
Adsorption isotherm of CATA on steel (*E* = −0.30 V) from 2.0 M HCl solution.

Based on the EIS results, data were obtained on the dependence of the surface coverage of steel by CATA on its concentration in the aggressive medium (Figure 5). At intermediate degrees of surface coverage by the organic compound, the adsorption of CATA can be described by the Temkin isotherm. The adsorption energy of CATA (−Δ*G_ads_*) on steel, calculated from the Temkin equation, is 54 kJ mol^−1^. Since it is known that in the case of chemisorption of substances on metals from aqueous solutions the free energy of adsorption (−Δ*G_ads_*) exceeds 40 kJ mol^−1^ [59], it can be concluded that the interaction of CATA molecules with the steel surface is of a chemical nature.

### 3.5. Kinetics of Cathodic Hydrogen Evolution and Hydrogen Penetration into Steel in the Presence of Corrosion Inhibitors

Using the bipolar electrode method, cathodic and anodic polarization curves, as well as the dependence of hydrogen penetration rates into steel on potential, were obtained in 2 M HCl solutions containing CATA and CrATA. This method allows both qualitative and quantitative evaluation of the effectiveness of various organic compounds in influencing the electrode reactions of the metal.

**Figure 6:**
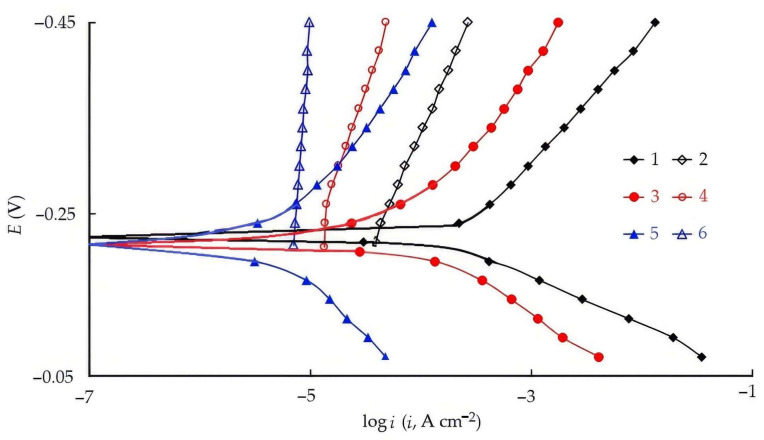
Cathodic and anodic polarization curves for steel (1, 3, 5) and the dependence of hydrogen penetration rate on potential (2, 4, 6): 2 M HCl (1, 2), 2 M HCl + 10 mM CrATA (3, 4), 2 M HCl + 10 mM CATA (5, 6).

As shown in Figure 6, both compounds, when added to 2 M HCl, significantly inhibit the cathodic and anodic processes as well as the penetration of hydrogen into steel. The presence of CrATA decreases the rates of cathodic hydrogen evolution and hydrogen permeation by several times, whereas CATA provides a much stronger effect, suppressing these processes by several orders of magnitude. The influence of CATA on the anodic dissolution of steel is also considerably more pronounced compared to CrATA, which highlights its superior inhibiting performance. These findings are consistent with the polarization measurements (Section 3.3), where CATA exhibited a simultaneous retardation of both cathodic and anodic reactions, and with the EIS data (Section 3.4), which confirmed the formation of protective adsorption layers on the steel surface.

### 3.6. Calculation of the Kinetic Rate Constants of the Main Stages of Cathodic Hydrogen Evolution and Penetration into Steel

The IPZ-analysis method [46,47] can be applied both in blank solutions and in solutions containing corrosion inhibitors, provided that the mechanism of cathodic hydrogen evolution remains unchanged. The slopes of the linear regions of the cathodic polarization curve and the dependence of lg iₚ on potential (Figure 6) must satisfy the condition

(19) $$\left|\frac{dE}{d\;\mathrm{lg}\;i_{c}}\right|<\;\left|\frac{dE}{d\;\mathrm{lg}\;i_{p}}\right|<\;2\left|\frac{dE}{d\;\mathrm{lg}\;i_{c}}\right|,$$

**Figure 7:**
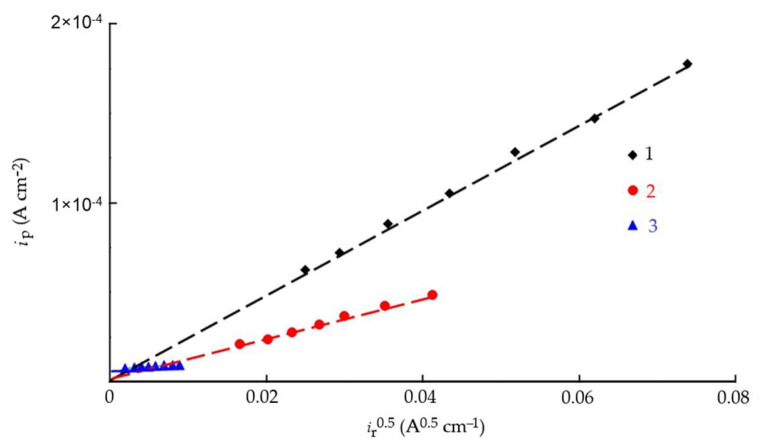
Dependence of the hydrogen permeation current through the membrane on the rate of its chemical recombination in 2.0 M HCl (1), in the presence of 10 mM CrATA (2), and 10 mM CATA (3).

In acidic media, the cathodic hydrogen evolution reaction on iron and steel proceeds via a discharge–chemical recombination mechanism, either with mixed control of the rate-determining step or through a slow discharge followed by an irreversible chemical recombination [46,47,60]. Relation (19) is one of the indicators of these mechanisms. Another indicator is the linear dependence of iₚ on the square root of the recombination rate (iᵣ)^1^ᐟ^2^ (Figure 7), which passes through the origin. As can be seen, both conditions are satisfied for the blank solution and for CrATA; therefore, the IPZ method can be applied for calculations [47]. In contrast, the addition of 10 mM CATA to 2 M HCl leads to a change in the mechanism of cathodic hydrogen evolution and hydrogen penetration into the metal. This effect is likely associated with the formation of dense protective inhibitor layers of CATA on the steel surface. CrATA, by contrast, does not form such layers.

By comparing the experimental curves of $\frac{dE}{d\log i_{c}}$ and $\frac{dE}{d\log i_{p}}$ (Figure 6) and applying the IPZ analysis method [46,47], the transfer coefficients of the hydrogen ion discharge reaction (α) were calculated for the background solution and the solution containing 10 mM CrATA.

When both hydrogen atoms and inhibitor particles are adsorbed on the metal surface, the determination of the rate constants of the main stages of hydrogen evolution and permeation into the metal by the IPZ method requires knowledge of the degree of surface coverage by the inhibitor (*θ_in_*). The steady-state values of *θ_in_* were determined from the ratio of *i_c_* values in the solutions with and without inhibitor [61].

Using the obtained α and *θ_in_* values, and assuming *r*_1_ = 0.3 [62], the rate constants of hydrogen ion discharge at potentials *E_i_* = −0.3 and −0.4 V (*k*_1,*i*_) and the kinetic–diffusion constants (*k*) were calculated in accordance with Equation (15). Based on the obtained *k* values and assuming the stationary hydrogen diffusion coefficient in the membrane D = 7.3 × 10^−5^ cm^2^/s [63], the rate constants of the chemical recombination of hydrogen atoms (*k*_r_) were determined using Equation (14) (Table 7).

Using the rate constants of the main stages of cathodic hydrogen evolution and permeation into steel, together with Equation (15), the degree of hydrogen coverage of the cathodically polarized steel surface (*θ*_H_) at E = −0.3 and −0.4 V was calculated for the background hydrochloric acid solution and in the presence of CrATA (Table 7).

For the calculation of subsurface hydrogen concentration in steel ($C_{H}^{s}.$), Equations (12) and (13) were applied, and the averaged values are presented in Table 7. The degree of protection of steel against hydrogen uptake ($Z_{H}^{s}.$) was calculated using

(20) $$Z_{H}^{s}.=[(C_{H}^{s}.\;-\;{C_{H}^{s}}_{,in})C_{H}^{s}.^{-1}]100\%,$$

where $C_{H}^{s}.$ and ${C_{H}^{s}}_{,in}$ are the subsurface concentrations of diffusible hydrogen in the electrolyte in the absence and in the presence of the inhibitor, respectively.

As seen, the addition of CrATA to the hydrochloric acid solution reduces the rate constant of H^+^ ion discharge and increases the kinetic-diffusion constant *k*. As a result, the hydrogen concentration in the metal phase decreases (Table 7 and Table 8) across the entire studied potential range. The degree of protection of mild steel against hydrogen uptake under cathodic polarization in the presence of 10 mM CrATA ranges from 81.6 to 88.2%.

When CrATA is added to 2 M HCl near the corrosion potential (*E* = −0.3 V), the amount of hydrogen adsorbed by the metal (*θ*_H_) slightly increases. This may be due to the inhibition of surface diffusion of hydrogen atoms upon adsorption of inhibitor particles on the metal (the hydrogen recombination constant, *k_r_*, decreases). When shifted to more cathodic potentials (*E* = −0.4 V), *θ*_H_ decreases because the rate of H^+^ ion discharge decreases, and this effect is much stronger than the decrease in the hydrogen recombination rate.

CrATA is an effective inhibitor not only of corrosion but also of hydrogen absorption in steel, since it decreases the H^+^ ion discharge rate constant (*k*_1,*i*_) and increases the kinetic-diffusion constant (*k*), thereby altering the relationship between *θ*_H_ and $C_{H}^{s}$. This effect is likely associated with the inhibitor blocking hydrogen absorption sites and hindering the transfer of hydrogen atoms from the surface into the metal phase. CATA demonstrates even higher efficiency in reducing hydrogen penetration into steel. However, its influence on this process has a deeper nature and involves altering the mechanism of hydrogen evolution on the metal, which prevents a straightforward analysis of the kinetic parameters of this process.

### 3.7. AFM Study of Steel Surface

AFM micrographs of the steel surface before and after exposure to the acid are presented in Table 9.

In all cases, the presence of sludge on the surface was observed, with the highest amount found on samples immersed in the uninhibited solution. Exposure to 2 M HCl without any inhibitor for 24 h caused severe etching of the metal surface. Height variations exceeded 1 μm, and the root-mean-square roughness increased up to 142 nm. The work function of electrons slightly decreased compared to baseline values, which is attributed to the destruction of the surface oxide layers.

The presence of CrATA and CATA inhibitors in 2 M HCl significantly slowed down the degradation of the steel surface. On metal surfaces exposed to inhibited solutions, only isolated cracks and cavities were observed, with approximately 80% of the surface remaining undamaged. Topographical analysis indicates that the surface of the sample exposed to the acid solution containing CATA was less damaged. The substantially lower root-mean-square roughness and higher work function suggest that CATA is more effective in slowing steel corrosion in 2 M HCl, likely due to the formation of a protective film on the steel surface.

### 3.8. Layers Formed by CATA on Steel

**Figure 8:**
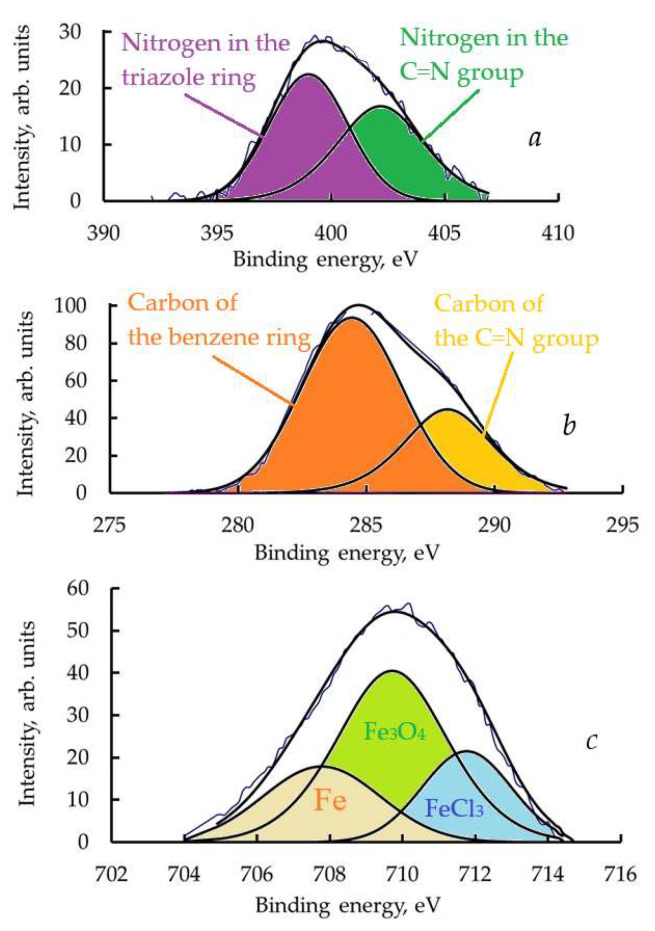
XPS spectrum of (**a**) N1s, (**b**) C1s and (**c**) Fe2p electrons.

XPS analysis of the steel surface after exposure to CATA-inhibited HCl solutions showed that a thin layer of organic material, chemically bonded to the metal, forms on the surface. This layer is not removed even after thorough washing in HCl solution with ultrasonic treatment. On the XPS spectra of the steel surface, this is evidenced by the appearance of the N1s peak corresponding to the nitrogen atoms in CATA molecules. Figure 8a shows the XPS spectrum of N1s electrons after the sample was kept at 20 °C for 2 h and then washed in HCl solution. In this spectrum, the peak at a binding energy of 399.5 eV corresponds to nitrogen in the triazole ring, while the peak at higher binding energy corresponds to nitrogen in the C=N group.

On the C1s XPS spectrum of the organic protective layer obtained under the same conditions (Figure 8b), two peaks can also be observed at binding energies of 285.0 eV and 287.2 eV, which are attributed to the carbon of the benzene ring and the C=N group.

The thickness of the organic layer present on the steel surface is relatively small, on the order of 2–3 nm, as indicated by the presence of metallic iron lines in the Fe2p electron spectrum shown in Figure 8c. In addition to the metallic iron peak, the spectra also show peaks corresponding to oxidized iron and FeCl_3_. These compounds, primarily iron oxides, form an intermediate layer between the metal and the organic part of the protective layer.

**Figure 9:**
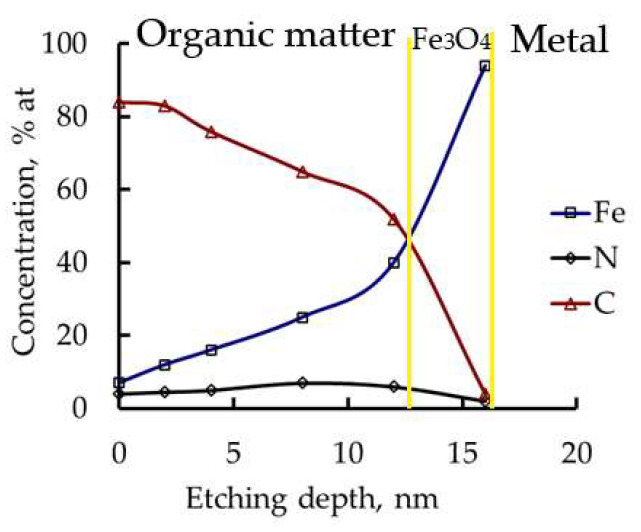
Depth profile of the protective film components (Fe, N, C) on the steel surface after exposure to CATA-inhibited HCl solution at 95 °C.

With increasing temperature, the nitrogen content in the chemisorbed layer decreases, while the carbon content increases; the thickness of the protective layer also grows. Figure 9 shows the distribution of chemical elements across the depth of the protective layer for samples after exposure to HCl at 95 °C. As can be seen, the protective layer at 95 °C is significantly thicker than at room temperature. Table 10 presents the composition and thickness of the protective layers for different exposure temperatures in HCl solution.

Analysis of the data presented in Table 10, together with the XPS spectra, indicates that at elevated temperatures the chemical composition of the organic component of the protective layer undergoes significant changes. These changes are likely associated with the hydrolysis of CATA molecules and subsequent chemical transformations of both CATA and its hydrolysis product, CA, resulting in the formation of a densely cross-linked polymeric layer on the steel surface. Examination of samples exposed to inhibited solutions without subsequent washing revealed that, atop the polymeric organic layer, there exist layers of physically adsorbed CATA molecules and potentially low-molecular-weight products of its chemical transformation, with a total thickness not exceeding 2–3 nm. This physically adsorbed layer is readily removed through ultrasonic cleaning.

The proposed mechanism of protective film formation by CATA involves initial chemisorption of the inhibitor onto the steel surface, followed by in situ chemical polymerization. Increasing temperature promotes partial hydrolysis of CATA, liberating CA and TA. The highly water-soluble TA desorbs into the aqueous medium, whereas CA remains incorporated in the organic layer, participating in polymer formation with the CATA molecules. Consequently, the relative carbon content of the organic portion of the protective layer increases with temperature, while the nitrogen content decreases. Notably, CA alone is capable of forming a protective film on steel in HCl solutions.

The formation of a chemisorbed polymeric layer by CATA on the steel surface results in pronounced inhibition of both steel corrosion and hydrogen absorption. Effective protection under high-temperature corrosive conditions (t ≥ 80 °C) is achieved only when a polymeric layer is chemically bound to the metal surface. Two principal pathways can lead to the formation of such protective layers: chemical transformation of the inhibitor into a polymeric compound and formation of a complex polymeric network [64]. In the present study, the first pathway is realized. CATA molecules chemisorb onto the steel surface, ensuring robust adhesion of the developing protective film. Furthermore, the presence of unsaturated bonds within the CATA molecules facilitates polymerization within the adsorbed organic layer, contributing to the formation of a coherent, dense, and highly protective surface film.

It is of particular importance to compare the protective performance of the newly developed corrosion inhibitor, CATA, with that of corrosion inhibitors for steels in hydrochloric acid solutions reported in recent years [12,13,14,15,16,17,18,19,20,21] (Table 1). Under the most aggressive experimental conditions (t = 100 °C), the addition of 10 mM CATA provides an inhibition efficiency of 99.6%, which considerably exceeds the values reported for the compounds listed in Table 1, even though those data were obtained at lower temperatures.

### 3.9. Analysis of the Condensation Products of Cinnamaldehyde with Amitrole

**Figure 10:**
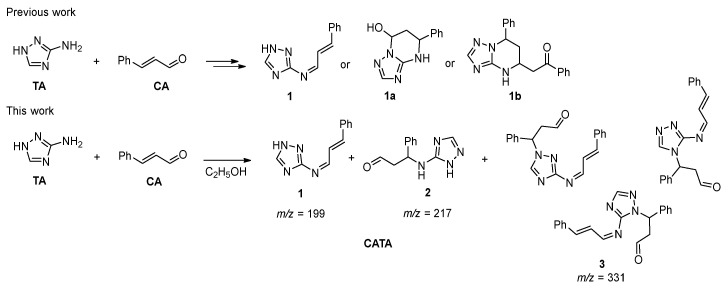
Products of the reaction of amitrole (3-amino-1,2,4-triazole) with cinnamaldehyde reported in references [66,67] and in this work.

For a more comprehensive discussion of the inhibition mechanism, it is essential to gain a thorough understanding of the structure of the resulting mixture of compounds (Figure 10). Since the inhibitor represents a mixture rather than a single substance, chromatographic-mass spectrometry was selected as the method for its identification.

Before analyzing the experimental data, it is useful to consider previously reported reaction products of aminotriazole (TA) with α,β-unsaturated aldehydes. The addition of amines to α,β-unsaturated aldehydes can proceed via two pathways—1,2- or 1,4-addition—depending on the nature of the reagents and the presence of catalysts [43].

Specifically, the reaction of 3-amino-1,2,4-triazole with unsaturated aldehydes has been investigated in two studies, both using cinnamaldehyde. According to the study by [65], heating equimolar amounts of the reagents in isopropanol or DMF leads to the formation of Schiff base products (azomethines). In contrast, when acetone was used as the solvent with piperidine as an additive, a cyclized product was obtained. In the work by Zemlyanoy [66], it was shown that, in the presence of morpholine and under heating, the formation of the 3b compound is also possible.

**Figure 11:**
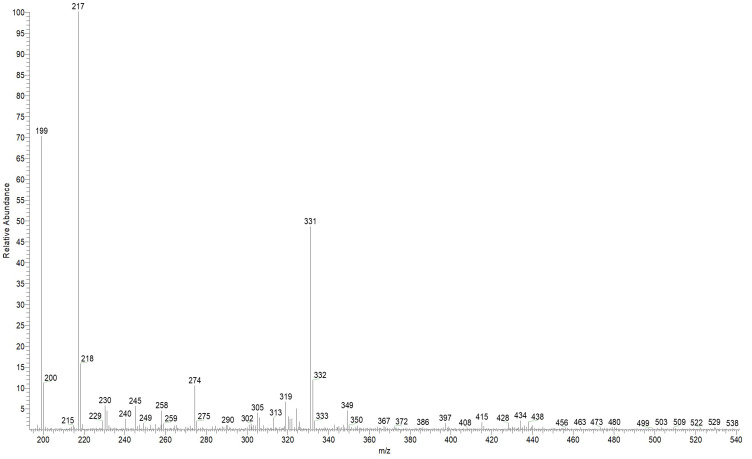
ESI mass spectrum of the reaction products (CATA) of amitrole (TA) with cinnamaldehyde (CA).

The ESI mass spectrum in the positive ion detection mode of the reaction products is shown in Figure 11. Among the most intense signals attributable to probable reaction products, ions at *m*/*z* 199, 217 and 331 can be distinguished. These signals correspond to protonated molecules with the following compositions: C_20_H_18_N_4_O for *m*/*z* 331, C_11_H_12_N_4_O for *m*/*z* 217, and C_11_H_10_N_4_ for *m*/*z* 199.

To confirm the proposed molecular formulas, as well as to elucidate the structures of these products, tandem mass spectrometry with collision-induced dissociation (CID) was employed.

**Figure 12:**
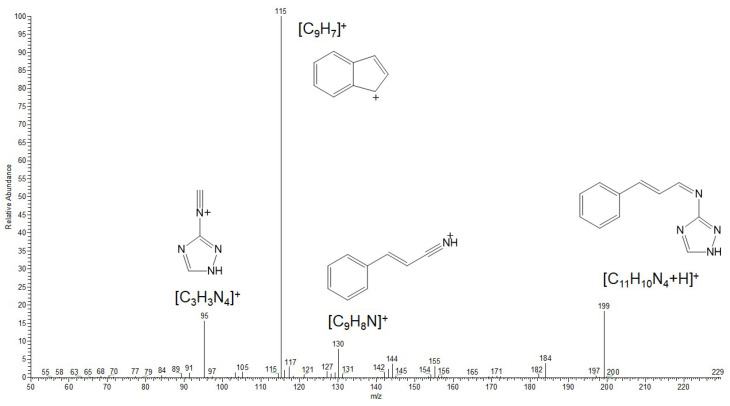
CID mass spectrum of the ion at *m*/*z* 199.

The analysis of the CID mass spectrum of the ion at *m*/*z* 199 (Figure 12) revealed that its structure corresponds to the 1.2-addition product. The most intense fragment ion at *m*/*z* 115 can be attributed to the cleavage of the TA moiety with the positive charge retained on the aromatic hydrocarbon fragment (indenyl cation). The expected protonated amitrole (TA) ion (*m*/*z* 85) was not detected; instead, a fragment at *m*/*z* 95 (C_3_H_3_N_4_) was observed. This finding indicates a stronger linkage between the cinnamaldehyde and amitrole fragments via a C=N double bond, consistent with the structure of the 1.2-addition product.

**Figure 13:**
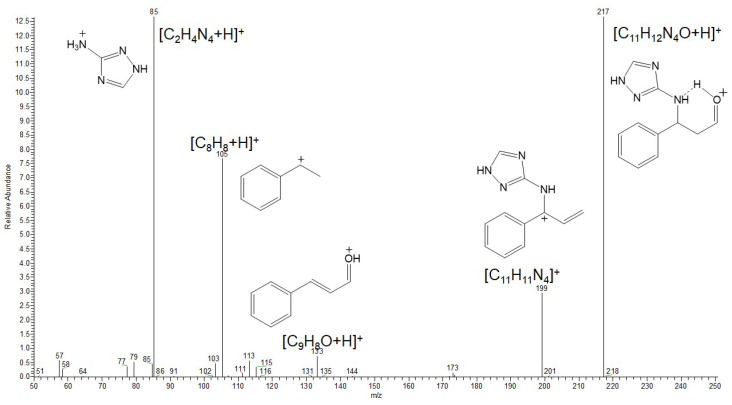
CID mass spectrum of the ion at *m*/*z* 217.

The CID mass spectrum of the ion at *m*/*z* 217 (Figure 13) shows that the primary fragmentation pathway corresponds to the loss of a water molecule (H_2_O). This observation suggests the presence of either a hydroxyl or an aldehyde group, the latter being capable of water elimination upon protonation. A prominent fragment at *m*/*z* 85 corresponds to the protonated amitrole molecule. Additionally, the presence of a protonated cinnamaldehyde ion (*m*/*z* 133) in the spectrum excludes the formation of more complex cyclic products. Therefore, the molecular formula C_11_H_12_N_4_O for the ion at *m*/*z* 217 can be assigned to the 1.4-addition product.

**Figure 14:**
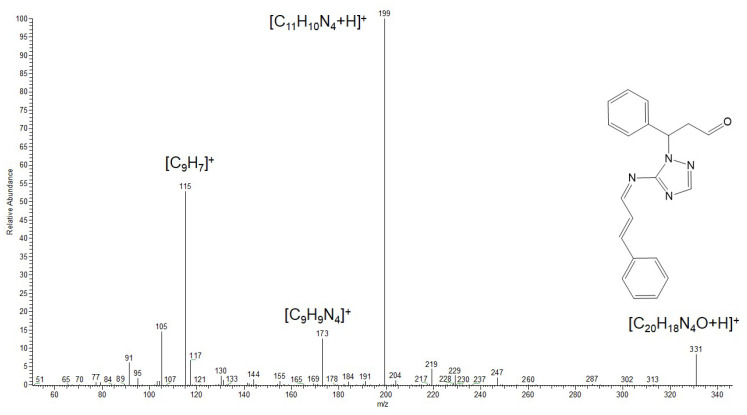
CID mass spectrum of the ion at *m*/*z* 331.

In the CID spectrum of the ion at *m*/*z* 331 (Figure 14), the formation of the fragment ion at *m*/*z* 199 corresponds to the loss of a cinnamaldehyde molecule during fragmentation, indicating the presence of a probable double-addition product. A closer examination of the remaining fragment ions in the spectrum further supports the assignment of this species to a mixed 1.2- and 1.4-addition product.

Thus, the analysis of the reaction products indicates that the main species formed are compounds of 1.2-, 1.4-, and mixed-type addition of cinnamaldehyde (CA) to the aminotriazole (TA) molecule. Two out of the three products (those retaining the C=C double bond) preserve the ability to undergo polymerization. Both the 1.2-addition and the mixed-addition products are susceptible to hydrolysis along the C=N bond, which explains the decrease in nitrogen content on the steel surface observed in the preceding sections. Based on the mass spectrometric analysis, it can be concluded that CATA represents a mixed inhibitor composed of three principal condensation products. It is important to emphasize that the reaction products formed under mild conditions do not include novel cyclic systems, which are resistant to hydrolysis and polymerization. Therefore, the synthesis of CI derived from unsaturated aldehydes and amines, particularly bifunctional ones, should preferably be carried out under mild conditions.

### 3.10. Molecular Dynamics of 1.2 and 1.4 Condensation Products on Metal Surface

**Figure 15:**
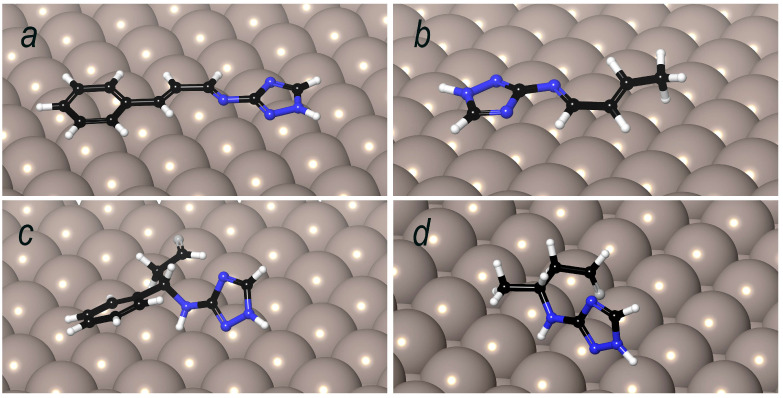
Adsorption models of the reaction products on the iron surface. Water molecules are omitted for clarity. (**a**) Product of 1.2-addition of cinnamaldehyde to aminotriazole. (**b**) Product of 1.2-addition of crotonaldehyde to aminotriazole. (**c**) Product of 1.4-addition of cinnamaldehyde to aminotriazole. (**d**) Product of 1.4-addition of crotonaldehyde to aminotriazole.

To evaluate the sorption properties of the 1.2 and 1.4 condensation products, molecular dynamics simulations were performed using the Forcite module of the BIOVIA Materials Studio 2023 software package. Figure 15 presents the interaction models of molecules corresponding to the 1.2-addition products (A, B) and the 1.4-addition products (C, D) of CA and CrA to amitrole (TA).

Based on the obtained adsorption models, it can be concluded that the most favorable orientation for surface coverage is adopted by the 1.2-addition products (A, B). This effect can be rationalized by the structural features of the molecules. In the 1.2-addition product of cinnamaldehyde with aminotriazole, all atoms are in sp^2^ hybridization, allowing the molecule to adopt a fully planar conformation and to align parallel to the iron surface. The 1.2-addition product of crotonaldehyde exhibits a nearly identical model, except that the methyl group lacks π-bonding and the corresponding carbon atom is in sp^3^ hybridization.

In contrast, the 1.4-addition products cannot adopt a planar conformation due to the presence of sp^3^-hybridized atoms. As a result of the additional rotational degrees of freedom, the 1.4-addition products have more difficulty achieving geometries favorable for adsorption on the iron surface. Therefore, based on the structural analysis of the 1.2- and 1.4-addition products in combination with molecular dynamics simulations, it can be concluded that the most effective corrosion inhibitors are expected to be the 1.2-addition products.

### 3.11. Molecular Orbital Calculations for the 1.2- and 1.4-Addition Products of Cinnamaldehyde with Aminotriazole

In studies on corrosion inhibitors, it is standard practice to compute the molecular orbitals of inhibitor molecules to rationalize or predict their adsorption efficiency and overall performance. The spatial distribution of molecular orbitals critically affects the interaction of organic molecules with metal surfaces. Specifically, the HOMO (highest occupied molecular orbital) can act as an electron donor to vacant orbitals on the metal atoms, whereas the LUMO (lowest unoccupied molecular orbital) may facilitate back-donation of electron density from the metal surface to unoccupied regions of the organic molecule.

**Figure 16:**
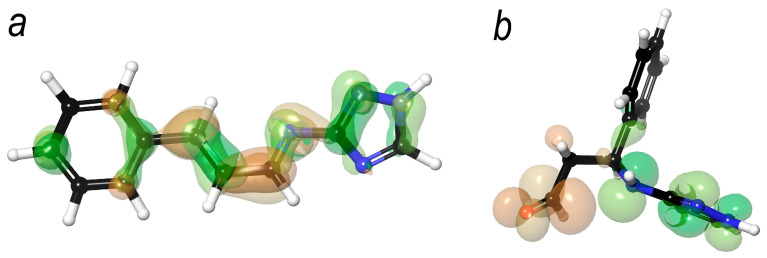
Distribution of HOMO (green) and LUMO (orange) orbitals in the cinnamaldehyde–aminotriazole adducts. (**a**) 1.2-addition product. (**b**) 1.4-addition product.

Integration of molecular orbital analysis with adsorption models obtained from molecular dynamics simulations enables a more precise assessment of inhibitor efficacy. When the HOMO and LUMO are localized in regions that directly interact with the metal surface, stronger and more effective electronic coupling is anticipated. In this study, we performed HOMO and LUMO calculations for the 1.2- and 1.4-addition products of aminotriazole with cinnamaldehyde, providing insight into their potential adsorption behavior and inhibitory efficiency (Figure 16).

For the 1.2- and 1.4-addition products, the HOMO is primarily localized in the triazole moiety. Differences are observed in the LUMO distribution: in the 1.2-addition product, the LUMO is concentrated along the double bonds connecting the triazole and phenyl fragments, whereas in the 1.4-addition product, the LUMO is localized in the aldehyde group. Combined with the adsorption models obtained in the previous stage, these results indicate that the 1.2-addition product should exhibit superior adsorption on the metal surface, as the molecule is planar and both HOMO and LUMO can participate in binding without conformational changes. The calculated HOMO–LUMO energy gaps are 3.8 eV and 5.1 eV for the 1.2- and 1.4-addition products, respectively. According to conventional criteria, a gap of 5–5.5 eV is considered favorable for corrosion inhibitor molecules, which would suggest that the 1.4-addition product could act as a more effective inhibitor [67]. However, the HOMO–LUMO gap also correlates with molecular reactivity: the smaller the gap, the higher the reactivity. Considering this, the 1.2-addition product is expected to be more reactive, for example, in polymerization reactions, which is advantageous in the present context. Therefore, given the mechanism of surface protection based on polymer formation, the conventional 5–5.5 eV criterion is not directly applicable to this system.

## 4. Conclusions

The condensation product of cinnamaldehyde and 3-amino-1,2,4-triazole, herein referred to as CATA, exhibits highly effective corrosion inhibition for mild steel in 2 M HCl over the temperature range of 20–100 °C. The increase in protective efficiency of CATA with temperature, along with the extremely low corrosion rates in its presence, classifies it as a high-temperature corrosion inhibitor.

The high efficiency of CATA in protecting mild steel in HCl solutions results from the strong inhibition of the metal’s electrode reactions by this compound. Adsorption of CATA on mild steel conforms to the Temkin isotherm, with a calculated Gibbs free energy of adsorption (−Δ*G_ads_*) of 54 kJ mol^−1^, indicating chemisorption between the steel surface and CATA molecules.

The addition of CATA markedly reduces hydrogen penetration into mild steel. Kinetic analysis indicates that its effect is sufficiently strong to alter the mechanism of the cathodic reaction compared to crotonaldehyde-based condensation products. This effect is attributed to the formation of a protective inhibitor film on the steel surface.

The inhibition properties of CATA suggest the formation of a protective inhibitor layer on the steel surface during corrosion in acidic solutions. This hypothesis is supported by XPS analysis, which reveals an organic protective layer of 2–12 nm thickness depending on temperature.

Mass spectrometric analysis of the reaction between cinnamaldehyde and 3-amino-1,2,4-triazole revealed the formation of 1,2- and 1,4-addition products, along with a double-addition compound. Molecular dynamics simulations indicated that the 1,2-addition product exhibits stronger adsorption on the iron surface due to its planar geometry, which enables closer contact with the metal. Considering its electronic structure and polymerization ability, the 1,2-addition product is likely the main active component responsible for the inhibition efficiency of the CATA mixture.

## Figures and Tables

**Table 1:** Protection of steels in HCl solutions with various corrosion inhibitors.

№	Inhibitor	Corrosive Environment	Steel	*Z*, %	Ref.
1	Nitrogen doped carbon dots synthesized using a dopamine (400 ppm)	1 M HCl (25–55 °C)	Q235 carbon steel	86.2–92.9	[12]
2	(S,E)-2-((1-(4-Minophenyl)ethylidene)amino) (3-(4-hydroxyphenyl)propanoic acid) (10 mM)	1 M HCl (30 °C)	Carbon steel	90.4	[13]
3	2-Cyano-N-(6-methylbenzo[d]thiazol-2-yl)-3-(4-nitrophenyl) acrylamide (0.1 mM)	2 M HCl (25–45 °C)	Carbon steel	73.9–85.8	[14]
4	*bis*-Azo dyes derived from 1,5-dihydroxynaphthalene (1 mM)	1 M HCl (24 °C)	Carbon steel	78.2–97.1	[15]
5	1-Dodecyl-2-((dodecylthio)methyl)-1Hbenzimidazole (1 mM)	1 M HCl (30–60 °C)	Carbon steel	71.1–95.4	[16]
6	*Potentilla erecta* (Tormentil) extract (300 ppm)	1 M HCl (25–55 °C)	360 carbon steel	90.2–92.7	[17]
7	*Dillenia suffruticosa* leaves extract (1 g L^−1^)	1 M HCl (25 °C)	Mild steel	91.3	[18]
8	*Platanus acerifolia* leaf extract (0.4 g L^−1^)	1 M HCl (25–45 °C)	Q235 carbon steel	83.8–88.9	[19]
9	Spinach extract (0.3 g L^−1^)	1 M HCl (20–50 °C)	Q235 carbon steel	88.6–95.3	[20]
10	Sodium carbenicillin, sodium ampicillin, and sodium sulbactam (8 mM)	1 M HCl (25–40 °C)	Q235 carbon steel	49.1–96.8	[21]

**Table 2:** Composition, molar mass and labels of organic inhibitors tested.

№	Inhibitor	Molecular Formula	Molar Mass (g mol^−1^)	Structural Formula	Label
1	cinnamaldehyde	C_9_H_8_O	132	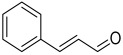	CA
2	benzaldehyde	C_7_H_6_O	106	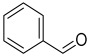	BA
3	crotonaldehyde	C_4_H_6_O	70	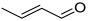	CrA
4	1H-1,2,4-Triazol-3-amine	C_2_H_4_N_4_	84	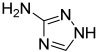	TA

**Table 3:** Influence of inhibitors (*C*_in_ = 10.0 mM) on the specific mass loss (Δ*m*^s^, g m^−2^)**,** corrosion rate (*W*, g m^−2^ h^−1^) and the degree of protection (*Z*, %) mild St3 steel in 2.0 M HCl.

Inhibitor	Δ*m*^s^, *W*, *Z*	*T* (°C)								
		20	30	40	50	60	70	80	90	100
–	Δ*m*^s^	3.6	8.6	18	45	120	290	700	1170	1830
	*W*	1.8	4.3	9.0	23	60	145	350	590	920
TA	Δ*m*^s^	2.2	4.0	7.6	20	68	170	450	1060	1770
	*W*	1.1	2	3.8	10	34	85	230	530	880
	*Z*	38.9	53.5	57.8	55.6	43.3	41.4	35.7	9.4	3.3
CA	Δ*m*^s^	0.86	1.6	2.6	6.2	12	16	30	49	340
	*W*	0.43	0.8	1.3	3.1	6	8	15	24.5	170
	*Z*	76.1	81.4	85.6	86.2	90.0	94.5	95.7	95.8	81.4
CATA	Δ*m*^s^	0.70	0.96	1.4	1.9	2.2	3.0	4.4	5.8	6.6
	*W*	0.35	0.48	0.70	0.95	1.1	1.5	2.2	2.9	3.3
	*Z*	80.6	88.8	92.2	95.8	98.2	90.0	99.4	99.5	99.6
CA + TA	Δ*m*^s^	0.20	0.54	0.84	2.0	5.2	9.8	12	18	130
	*W*	0.10	0.27	0.42	1.0	2.6	4.9	6	9	65
	*Z*	94.4	93.7	95.3	95.5	95.7	96.6	98.3	98.5	92.9
CrA	Δ*m*^s^	3.4	8.2	10	16	30	53	170	690	1290
	*W*	1.7	4.1	5.0	8.0	15	26.5	85	345	645
	*Z*	5.6	4.7	44.4	64.4	75.0	81.7	75.7	41.0	29.5
CrATA	Δ*m*^s^	1.3	2.6	3.8	7.6	15	27	45	120	190
	*W*	0.65	1.3	1.9	3.8	7.5	13.5	22.5	60	95
	*Z*	63.9	69.8	78.9	83.1	87.5	90.7	93.6	89.7	89.6
CrA + TA	Δ*m*^s^	2.8	8.0	10	13	26	45	100	430	750
	*W*	1.4	4.0	5.0	6.5	13	22.5	50	215	375
	*Z*	22.2	7.0	44.4	71.4	78.3	84.5	85.7	63.2	59.0
BA	Δ*m*^s^	4.0	8.0	15	26	63	140	430	990	1630
	*W*	2.0	4.0	7.5	13	31.5	70	215	495	815
	*Z*	–11.1	7.0	16.7	42.2	47.5	51.7	38.6	15.4	10.9
BATA	Δ*m*^s^	2.4	5.0	8.8	16	34	92	240	460	1020
	*W*	1.2	2.5	4.4	8	17	46	120	230	510
	*Z*	33.3	41.9	51.1	64.4	71.7	68.3	65.7	60.7	44.3
BA + TA	Δ*m*^s^	2.2	4.8	4.6	18	42	100	260	600	1200
	*W*	1.1	2.4	2.3	9	21	50	130	300	600
	*Z*	38.9	44.2	74.4	60.0	65.0	65.5	62.9	48.7	34.4

**Table 4:** Effective activation energies of steel corrosion in HCl solutions.

Inhibitor	-	TA	BATA	CrATA	CATA	CA
*E*_ac_, kJ mol^−1^	74	81	70	57	26	52

**Table 5:** Corrosion parameters of spring steel in 2.0 M HCl.

Inhibitor	Temperature, °C	*W*, g m^−2^ h^−1^	*V*^s^(H_2_), mL (100 g of Metal)^−1^	*π*, %
-	25	11.4	4.62	- *
10 mM CA	25	1.3	0.75	100
10 mM CATA	25	0.73	0.60	100
-	60	48.7	2.56	-
10 mM CA	60	12.2	0.36	52
10 mM CATA	60	2.2	0.33	100

* complete loss of ductility.

**Table 6:** Potentials of corrosion *E*_cor_ (V), Tafel slopes *b_c_* and *b_a_* (V), densities of cathodic and anodic currents (*i_c_* and *i_a_* (A m^−2^)), degree of protection of cathodic and anodic reactions (Z_c_ and Z_a_ (%)) of mild steel in 2 M HCl in the presence of organic compounds at *E* = −0.300 and −0.150 V, respectively; *t* = 60 °C.

Inhibitor	*E*_cor_, V	*b*_c_	*b*_a_ *	*i*_c_	*i*_a_	*Z*_c_	*Z*_a_
2M HCl							
-	−0.235	0.130	0.060	866	872	-	-
0.03 mM CATA	−0.230	0.135	0.065	708	636	18.2	27.1
0.1 mM CATA	−0.225	0.135	0.070	382	266	55.9	69.5
1.0 mM CATA	−0.200	0.140	0.090	162	59.4	81.3	93.2
10.0 mM CATA	−0.260	0.180	*i_d_* **	8.7	15.0	99.0	98.3
10 mM TA	−0.235	0.140	0.065	605	588	30.1	32.6
10 mM CA	−0.240	0.180	0.120	56.1	84.5	93.5	90.3
10 mM CA + 10 mM TA	−0.245	0.180	0.100	24.6	30.5	97.2	96.5

* The Tafel slopes of the linear part of the anodic polarization curve nearest to *E_cor_* are given. ** *i_d_* is the limiting current.

**Table 7:** Values of kinetic constants, degree of surface coverage of steel by hydrogen atoms (*θ*_H_), subsurface concentration of diffusible hydrogen ($C_{H}^{s}$), and degree of protection of steel against hydrogen uptake ($Z_{H}^{s}$) under cathodic polarization (*E* = −0.3 V) of mild steel in HCl solution.

Solution	*k*_1,i_, mol cm^−2^ s^−1^	*k*, cm^3^ mol^−1^	*k*_r_, mol cm^−2^ s^−1^	*θ*_H_ × 100	$C_{H}^{s}$, mol cm^−3^	$Z_{H}^{s}$, %
2 M HCl	9.73 × 10^−9^	3.5 × 10^5^	7.5 × 10^−6^	3.43	1.0 × 10^−7^	
2 M HCl + 10 mM CrATA	2.18 × 10^−9^	2.1 × 10^6^	8.3 × 10^−7^	3.57	1.8 × 10^−8^	81.6

**Table 8:** Values of kinetic rate constants, surface coverage of the metal by hydrogen atoms (*θ*_H_), concentration of diffusible hydrogen ($C_{H}^{s}$), and the degree of protection of steel against hydrogen absorption ($Z_{H}^{s}$) under cathodic polarization (E = −0.4 V) for low-carbon steel in HCl solution.

Solution	*k*_1,i_, mol cm^−2^ s^−1^	*k*, cm^3^ mol^−1^	*k*_r_, mol cm^−2^ s^−1^	*θ*_H_ × 100	$C_{H}^{s}$, mol cm^−3^	$Z_{H}^{s}$, %
2 M HCl	7.17 × 10^−8^	3.5 × 10^5^	7.5 × 10^−6^	9.38	3.17 × 10^−7^	-
2 M HCl + 10 mM CrATA	9.64 × 10^−9^	2.1 × 10^6^	8.3 × 10^−7^	7.61	3.73 × 10^−8^	88.2

**Table 9:** AFM Study Results of Steel Surface.

Treatment	Micrograph 600 × 400 μm	Topography 30 × 30 μm	Root Mean Square Roughness, nm	Work Function, eV
Blank	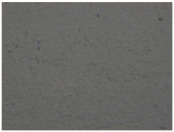	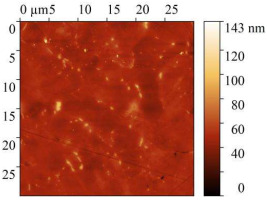	5.25 ± 1	4.96
2 M HCl	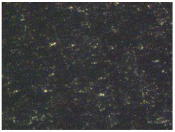	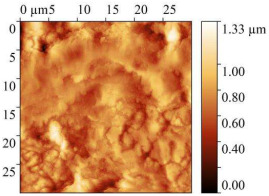	141.9 ± 10	4.73
2 M HCl + CrATA	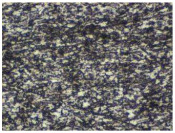	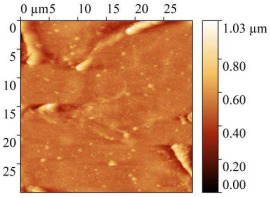	63.59 ± 5	5.9
2 M HCl + CATA	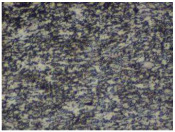	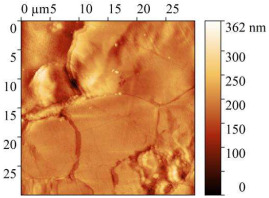	31.32 ± 3	5.63

**Table 10:** Chemical composition and thickness of protective layers formed on steel by CATA at different HCl exposure temperatures (metal samples after ultrasonic cleaning).

T, °C	C, %	N, %	O, %	Cl, %	Fe, %	Thickness, nm
20	32.4	11.3	9.1	5.2	21.3	2 ± 0.2
60	42.6	9.2	12.4	3.4	17.2	6 ± 0.5
95	63.9	5.4	8.4	2.1	12.3	12 ± 0.5

## Data Availability

The original contributions presented in this study are included in the article. Further inquiries can be directed to the corresponding authors.

## Abbreviations

The following abbreviations are used in this manuscript:

CA	cinnamaldehyde
BA	benzaldehyde
CrA	crotonaldehyde
TA	1H-1,2,4-Triazol-3-amine (aminotriazole)
CATA	condensation products of cinnamaldehyde with aminotriazole
BATA	condensation products of benzaldehyde with aminotriazole
CrATA	condensation products of crotonaldehyde with aminotriazole
